# Engineered Cell Microenvironments: A Benchmark Tool for Radiobiology

**DOI:** 10.1021/acsami.4c20455

**Published:** 2025-01-15

**Authors:** Qais Akolawala, Angelo Accardo

**Affiliations:** † Department of Precision and Microsystems Engineering, Faculty of Mechanical Engineering, Delft University of Technology, Mekelweg 2, 2628 CD Delft, The Netherlands; ‡ Holland Proton Therapy Center (HollandPTC), Huismansingel 4, 2629 JH Delft, The Netherlands

**Keywords:** engineered cell microenvironments, cancer, 3D printing, organ-on-a-chip, organoids, radiobiology

## Abstract

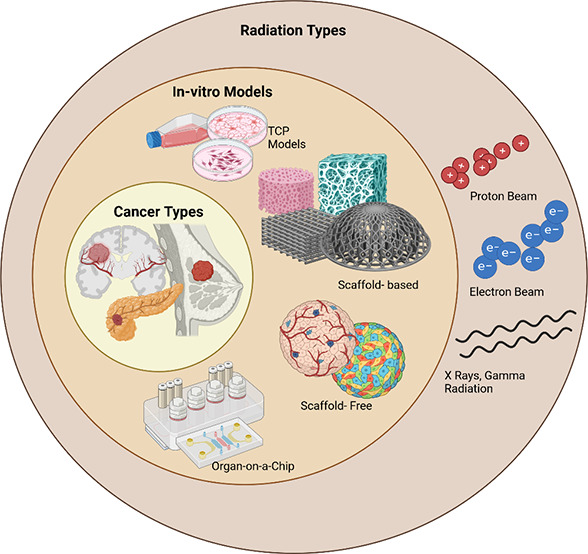

The development of engineered cell microenvironments for fundamental cell mechanobiology, in vitro disease modeling, and tissue engineering applications increased exponentially during the last two decades. In such context, in vitro radiobiology is a field of research aiming at understanding the effects of ionizing radiation (e.g., X-rays/photons, high-speed electrons, and high-speed protons) on biological (cancerous) tissues and cells, in particular in terms of DNA damage leading to cell death. Herein, the perspective provides a comparative assessment overview of scaffold-free, scaffold-based, and organ-on-a-chip models for radiobiology, highlighting opportunities, limitations, and future pathways to improve the currently existing approaches toward personalized cancer medicine.

## 1 Introduction

Radiobiology is a field of research that investigates the effects of ionizing radiation (e.g., X-rays/photons, high-speed electrons, and high-speed protons) on biological (cancerous) tissues and cells, in particular in terms of DNA damage leading to cell death.^1^ Systematic studies on the morphological and functional changes of cancer and healthy surrounding cells after being exposed to radiation cannot be routinely performed on animals due to their scarcity and ethical reasons or living tissues derived from biopsies as well due to their scarcity and the difficulty in preserving them alive for a long time. Proton, photon,^2^ and electron-beam^3,4^ radiation are the currently available main radiotherapy techniques for treating cancer. During the past decade, several clinical studies focused attention on the comparison between photon (the conventional X-ray radiation treatment) and proton (the more recent) therapy. Protons have a depth-dependent energy deposition,^5^ which is very different from that of X-rays. The low deposition of energy at the entrance of the tissue ensures that this region is not damaged and the beam retains its energy. At the Bragg peak, which is targeted at the tumor site, the maximum dose is deposited. Therefore, theoretically, the damaging effect of protons can be fundamentally much better targeted at the tumor, sparing the healthy surrounding tissue. This assumes particular relevance in light of recent advances concerning FLASH therapy^6^ (a technique based on the use of ultrahigh dose rates, maintaining the anticancer action of conventional radiation therapy but reducing induced damage to the healthy surrounding tissue). FLASH and conventional modalities feature respectively hundreds of gray per second and a few gray per minute dose rates. Nonetheless, a quantifiable comparative analysis of these treatments, including also electron-beam therapy^7^ or heavy ions,^8^ across different types of cancer types requires the creation of physiologically relevant, reproducible in vitro cancer models. There is therefore an urgent need for cell-instructive engineered microenvironments that can be exploited as standardized and biomimetic in vitro models for understanding how cancer cells’ development and response to radiotherapy take place in a configuration that overcomes the limitations of conventional cell monolayers provided by “petri-dish” approaches.

**Figure 1:**
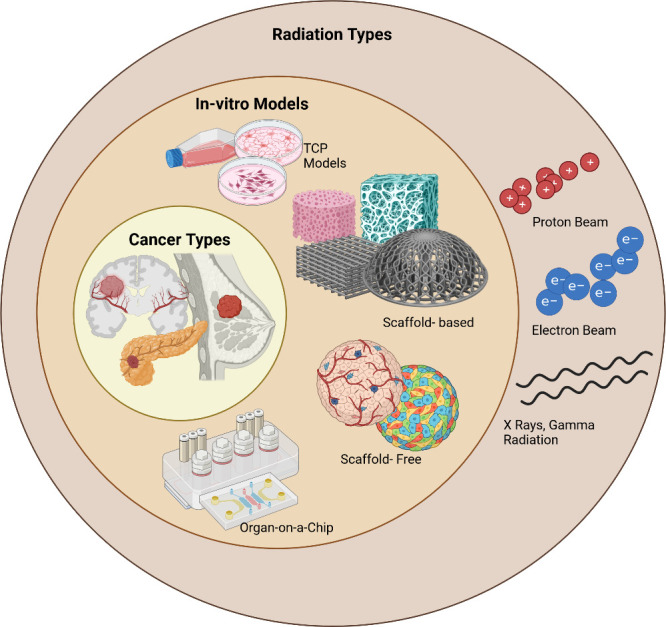
Schematic representation of the radiation sources and main cancer cell culture in vitro models for radiobiology: TCP, scaffold-based models, scaffold-free models, and organ-on-a-chip models. Created using Biorender.

One of the main targets of these models is to mimic as much as possible the native tumor microenvironment (TME), in terms of dimensional, geometric, biochemical, and mechanical features. In this Perspective, we discuss the advent of engineered cell microenvironments as a benchmark tool for radiobiology (Figure 1). In particular, we provide a comparative overview of the three main categories of available models, scaffold-free, scaffold-based and organ-on-a-chip, highlighting the latest developments in the field as well as advantages and disadvantages of each approach. Finally, we provide an outlook about the new pathways that we envision to further address the current needs to develop models enabling tangible personalized cancer medicine. Table 1 highlights the main findings, advantages, and disadvantages of each model category that are discussed in the following sections.

**Table 1:** Main Findings and Comparisons of the Reported In Vitro Model Categories Used for Radiobiology Studies

	scaffold-free 2D and 3D Models	scaffold-based 3D models	organ-on-a-chip models
findings	2D TCP models result in cell monolayers and are widely employed in all types of in vitro radiobiology experiments	(bio)printing and fabrication methods generate scaffolds for cells using many different biomaterials	fluid-flow and dynamical cell interactions are recreated
	spheroids and organoids are formed from the organic 3D self-assembly of cells	specific features of the natural ECM are reproduced for more representative radiotherapy studies	good approximation of the dynamics surrounding healthy and cancer cells in vivo is enabled
	formation of an oxygen gradient within 3D models is an important parameter for the study of radiation outcomes		
advantages	inexpensive, high throughput, and highly reproducible (2D)	highly reproducible and can maintain good quality control	can model long-term radiobiological effects in cells and tissues (weeks, months)
	high degree of cell–cell and cell–matrix interactions (3D)	integrable with microfluidics	can be used to create “compartments” and model different cell-specific functions simultaneously
	models tumor core and edge effects well (3D)	adaptable for specific evaluation techniques and readouts	
	expandable to multicellular models (2D and 3D)		
disadvantages	lacks 3D spatial tissue organization (2D)	requires cross-domain collaboration and expertise	development of a model is time-consuming before the experiment
	expensive and does not work effectively for all cell types (3D)	often requires specialized equipment, which can increase costs	practical considerations with beamlines are due to associated paraphernalia
	limited reproducibility (3D)	lacks a standardized approach for fabrication and evaluation in radiobiology	lower throughput when compared to other in vitro models
	no physiological fluid flow or pressure (2D and 3D)		
	limited imaging capabilities (3D)		

## 2 Scaffold-Free In Vitro Models for Radiobiology

### 2.1 Tissue Culture Plastic (TCP) Models

TCP models refer to the use of flasks, Petri dishes, and well-plates to culture and conduct experiments with cells. TCP are usually made of stiff materials such as polystyrene or polycarbonate (Young’s modulus *E* ≈ 2–4 GPa), are sterilizable, and are suitable for high-throughput analysis. Typically, TCP models employ a layer of biochemical coating (e.g., laminin, collagen, fibronectin, and Matrigel with a Young’s modulus ranging from a few pascal to hundreds of kilopascal) to favor cell adhesion. Nonetheless, such layers are typically submicrometric thick, and cells are known to probe stiffnesses until a few microns in depth;^9,10^ therefore, the Young’s modulus of the Petri dish must be taken into account when considering cell–substrate interactions. In the context of radiobiological studies, these flasks are typically used to perform clonogenic assays.^11,12^ Clonogenic assays are widely used to determine at which rate cells exposed to radiation continue to proliferate, the changes that occur in these subsequent clones, and the survival percentage of the cells. Even though TCP is inexpensive and easy to handle, it leads to the formation of unrealistic two-dimensional (2D) cell monolayers, which substantially differ from the three-dimensional (3D) spatial configuration of real cancer tissues. For this reason, in the context of radiobiology studies, they can provide results that do not align with in vivo^13,14^ or other 3D in vitro models,^15,16^ often resulting in higher DNA damage upon exposure to treatment.^17,18^ In experiments involving electron-beam therapy, TCP models also may lead to uneven dose distribution due to the unintended interaction between the radiation and the plastic.^7^ Despite these pitfalls, the TCP-based clonogenic assays are considered the gold standard but still need to be adapted to 3D cell culture.^19,20^

The use of 2D TCP cell models can lead to a loss of specific cell functionalities. Proteins, such as integrins, are responsible for adhesion of the cells to their surrounding environment. Studies have shown that the usage of a fibronectin^20^ coating on the plastic substrates leads to a higher expression of these integrins, and subsequently a higher surviving fraction of the cells. This is a phenomenon called cell-adhesion-mediated radioresistance.^20^ This effect is observed in many cancer types including breast, pancreas, lung, and glioblastoma and shows that the chemical composition of the extracellular matrix (ECM), and the use of these materials influences the radiosensitivity and thus the accuracy of the in vitro model.^20^ Cordes et al. exposed a fibronectin-coated 2D TCP-ECM-based model cultured with either glioblastoma, pancreatic cancer, lung carcinoma melanoma, normal human skin, or lung fibroblast cells to 240 kV X-ray radiation in a dose range of 0–8 Gy. They found that the fibronectin coating leads to an increase in cell adhesion, which, in turn, results in an increase in radioresistance compared to cells grown on only the polystyrene substrate.^20^ From a mechanobiology point of view, it is also known how substrate stiffness affects the morphology, proliferation, and radiosensitivity of cancer cells, as reported for cervical squamous carcinoma,^21^ where stiffer substrates promoted proliferation and increased radioresistance of cervical cancer cells by affecting PI3K/Akt apoptosis pathways.

The advantages of TCP models should not be underestimated, however. They are easy to use and reproducible and can be employed for a wide variety of biological end points. TCP models are often functionalized with matrix materials (such as collagen, laminin, or Matrigel) to promote cell adhesion^20^ and/or modulate parameters such as oxygen concentrations,^22^ which can play a role for the emulation of hypoxic environments, distinctive of cancer.^23^ Further, the monolayer model enables high-throughput immunofluorescence image-based analysis. The study of radiotherapy effects can often be extensive because numerous replicas are required for statistically robust results.^11^ Therefore, TCP models in the context of radiobiology studies, involving a large variety of parameters such as the type of radiation source (e.g., X-rays, protons, electrons, and heavy ions), dose rate (FLASH or conventional), and delivery method (single dose, fractionation, and continuous or pulsed delivery), can be helpful at the cost of lower physiological relevance. A large body of literature, information, and expertise on the handling of TCP models already exists, enabling the investigation of different aspects related to radiotherapy responses such as the effects of hyperthermia,^24^ oxygen concentration,^25^ or radiotherapy-based alterations of cell migration.^26^ The standard, inexpensive, reproducible, and high-throughput approach of TCP models also allows one to compare radiobiological research^25^ and reduce variability related to parametric studies involving changes in the radiation type, dose, and dose delivery.

### 2.2 Tumor Spheroid Models

**Figure 2:**
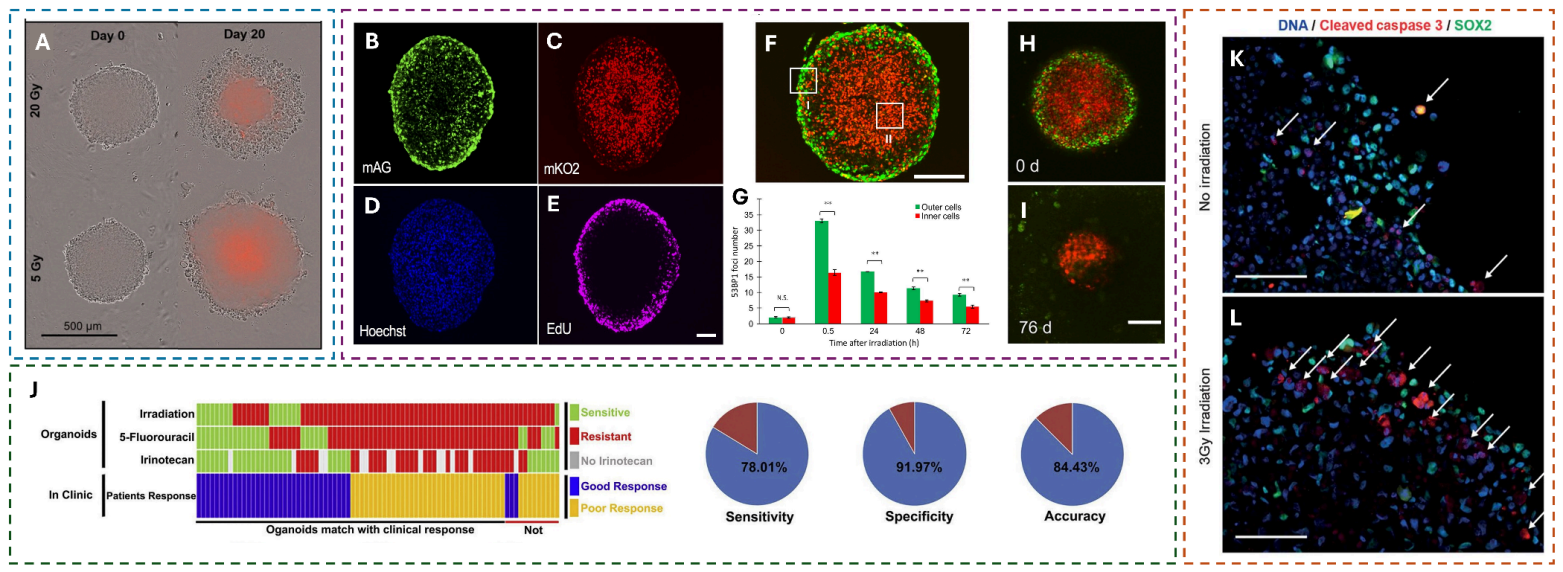
Examples of scaffold-free models and their responses to radiation. (A) Phase contrast images superimposed with propidium iodide (PI) fluorescence snapshots from time-lapse imaging of colorectal cancer spheroids irradiated with 20 or 5 Gy at 0 and 20 days after exposure. Scale bar: 500 μm. Adapted from ref (28). Available under CC-By 4.0. Copyright 2020 Springer Nature. (B–I) Human tongue squamous cell carcinoma spheroids stained using the Fucci method. Adapted from (29). Available under a CC-BY-NC-ND license. Copyright 2017 Cancer Science published by John Wiley & Sons Australia, Ltd. on behalf of Japanese Cancer Association. (B) Proliferating human tongue squamous cell carcinoma cells on the rim are stained fluorescent green. (C) Red cells in the quiescent stage are localized in the core of the spheroid. (D) Cell nuclei in the spheroid are stained with Hoechst. (E) Cells in the S-phase (synthesizing DNA) are marked with EdU in pink (in parts B–E, scale bar = 100 μm). (F) Overlay of the cells on the periphery and inside the tumor spheroid (scale bar = 200 μm). (G) 53BP1 DNA damage foci number at the indicated times after 10 Gy of irradiation. The green bar indicates cells on the periphery and the red bar those in the internal regions. (H and I) Growth of the spheroid over 76 days after being irradiated with 10 Gy. The inner quiescent cells remain in a smaller core, and the outer portion disintegrates (scale bar = 200 μm). (J) Correlation of rectal tumor organoid model outcomes with patient treatment outcomes. It is observed that if the organoid shows that any of the three treatments used is effective, then the patient outcomes correlate positively. Reproduced from ref (30). Available under a CC-BY license. Copyright 2019 Elsevier Inc. (K and L) SOX2 positive cells (green) are negative for cleaved Caspase-3 (red) after being exposed to 3 Gy radiation on a glioblastoma organoid rim. The arrows indicate the apoptotic cells positive for cleaved Caspase-3 (scale bars = 100 μm). Reproduced from ref (31). Copyright 2016 American Association for Cancer Research.

The organization of the cells and their interaction with the ECM lead to biophysical changes, which can mediate DNA damage, cell survival, proliferation, and even differentiation.^20^ Tumor spheroids are 3D tissue-like architectures resulting from spontaneous cell assembly, featuring cell–cell interactions, and reproducing physiological tumor conditions. Various methods can be employed to generate tumor spheroids including the hanging-drop method, bioreactors, rotational flasks, and, more recently, microfluidics.^27^ Cell aggregation leads to the formation of an oxygen gradient and a necrotic cellular core due to the difficult penetration of cell medium nutrients. This oxygen gradient allows one to study the effects of hypoxia on tumor growth kinetics. The hypoxic environment enables, for instance, the assessment of the differential response of tumor spheroids to FLASH and conventional radiotherapy by measuring their changes in mass or size after irradiation. In their work, Brüningk et al.^28^ formed monoculture cellular spheroids with various human cancer cell lines (colorectal cancer and squamous cell carcinoma) and exposed them to X-ray radiation doses of up to 20 Gy. As can be observed in Figure 2A, irradiated spheroids retained a dense structure with dead cells detaching from the outer cell layers [propidium iodide (PI) staining, allowing the visualization of dead cells], resulting in gradual shrinkage from the outside inward. The necrotic core of the spheroid is more compact in the 5 Gy spheroid compared to the 20 Gy one, and it is a result of the varying oxygen concentration between the core and rim of the spheroid. Samples irradiated with 10 and 20 Gy continuously shrank, preventing central necrosis and resulting in a decrease in the PI intensity due its dependence on the spheroid volume captured in the focal plane upon imaging.^28^ In this study, the authors also discuss the importance of 3D models because 2D models do not represent the physiological geometry of the tumors and may form an inaccurate basis to calculate the biologically equivalent dose to which patients are exposed to. 2D models provide an unrealistically uniform flow of oxygen and nutrients to the cells, which can affect their responses to the treatment modes being investigated. Furthermore, they also discuss the lack of a standardized method to quantify the spheroid response to radiation and that clonogenic analysis of the spheroid requires disaggregation of the spheroid, which may lead to nonrepresentative results.

Onozato et al.^29^ in their work conducted immunofluorescence imaging and analysis on spheroids obtained with human tongue squamous cell carcinoma cell lines (Figure 2B–I). The spheroids were created using human tongue squamous cell carcinoma (SAS) cells and exposed to X-ray radiation (130 kV, 0.75 Gy/min). The cells were fluorescently stained using Fluorescent Ubiquitination-Based Cell Cycle Indicator (Fucci). In this method, the cells change color from red to green as they progress through the cell cycle. They then compared to the radiosensitivity of proliferating cells (located at the spheroid periphery; green, Figure 2B) and nonproliferating ones (located in the spheroid core, under mild hypoxia; red, Figure 2C). Figure 2D shows the nuclei of the cells stained with Hoechst, and Figure 2E shows the cells that are synthesizing DNA (S-phase of the cell cycle) stained with 5-ethynyl-2′-deoxyuridine (EdU). These cells are fluorescently labeled and can be distinguished among each other in Figure 2F. Parts B and E of Figure 2 indicate that the proliferating cells are mostly localized at the periphery of the spheroid, while red cells form the quiescent core. Higher radioresistance was observed in the proliferating cells compared to 2D monolayers, after irradiation, due to the well-known contact effect that enhances cell radioresistance by cell–cell interaction.^32^ The rim cells showed a higher number of DNA damage foci compared to inner cells, which can be attributed to hypoxic conditions in the spheroid’s core. The bar graphs in Figure 2G highlights this difference in terms of 53BP1 foci formation, which is a protein immediately recruited by the cell to repair the induced DNA damage.^29^ Further, after irradiation, the spheroids were maintained in culture for 76 days, during which the outer proliferating layer is shed from the spheroid and the inner quiescent core remains intact. Figure 2H shows the spheroid immediately after irradiation, and Figure 2I shows the spheroid’s shrinkage after 76 days due to radiation-induced damage. It is also noteworthy that, when plated later into a 2D monolayer, the cells started to regrow and displayed clonogenicity.

Spheroids also present some disadvantages. Even though they have tissue-like features, they are affected by significant limitations such as the impossibility to maintain a uniform size^33^ and the lack of a vascular system.^34^ They therefore do not always represent the most physiologically relevant approach to study the radiation effects on cells. Generally, spheroid analysis after radiation necessitates its disaggregation to generate clonogenic survival assays. This leads to a loss of the TME, which contributes to the cellular radiation response. Onozato et al.^29^ demonstrated the difference between survival assays of spheroids and monolayers. In particular, they reported how spheroids at the end of the extended 76-day-long culture show the presence of a dormant quiescent core (Figure 2H), which is not observed in the presence of 2D monolayer clonogenic assays. In addition to this, it is worth mentioning that there were limitations related to the imaging of deeper regions of the spheroids (>100 μm from the surface), appearing dark due to the optical conditions of the employed confocal scanning microscopy system.^29^

### 2.3 Tumor Organoid Models

Organoids are 3D cell culture models in which a functional part of an organ is (minimalistically) recreated at the microscale in vitro. The most significant difference between spheroids and organoids is the use of multicellular models (i.e., embryonic-, adult-, induced pluripotent stem-cell-derived somatic cells along with tumor cells) to include a specific organ function or growth.^35,36^ Organoids feature also higher-order self-assembly structures compared to spheroids (which typically organize into spherical cellular aggregates) because stem cells self-organize through cell sorting and spatially defined differentiation to resemble organ cell types, structures, and functions.^36,37^ In order to foster cell assembly and organization of the tissue-like structures, synthetic and natural matrices are employed. Among these, we find Matrigel but also decellularized hydrogels,^38^ which feature relatively soft mechanical properties (Young’s modulus in the pascal to kilopascal range) playing a critical role in regulatory and pathological cell behaviors.^39^ Organoids are a promising model for radiobiological studies and have been employed to study the response of tumor and healthy cells to radiation doses for different types of cancer including glioblastoma,^31^ rectal,^30^ and pancreatic^40^ cancers. In their studies, Yao et al.^30^ and Pasch et al.^40^ show how rectal tumor organoids can be used to predict the response of the cancer to chemotherapy and radiation, where tumors are extracted from different patients requiring different doses of chemotherapy and radiotherapy in combination to be effective. Figure 2J shows the correlation of tumor organoid data to clinical patient outcomes from the study of Yao et al.^30^ and reports how a good clinical outcome was observed when the organoids were responsive to at least one of the three treatment components. Hubert et al.,^31^ on the other hand, created glioblastoma organoids from tumors derived from patient resections. The organoid has a unique feature, which allows the growth of cancer stem cells (CSCs) and nonstem cells simultaneously. In their work, they exposed the organoid to 3 Gy of X-rays and were able to observe a higher radioresistance in the CSCs compared to the nonstem cells around the organoid rim. The arrows in Figure 2K,L indicate the apoptotic cells, which were almost exclusively negative for the CSC marker SOX2. The preservation of such cellular heterogeneity makes organoids capable of better recapitulation of the in vivo tumor response to radiation compared with other 2D models. The formation of organoids from direct patient sources, in particular, enables the investigation of differential responses of healthy, tumor, and cancer stem cells and forms a promising tool for personalized medicine.^41^ One of the limitations of the organoid approach is, on the other hand, that they often require ECM-derived matrices to support their growth, such as Matrigel,^42^ which suffers from batch-to-batch variability and is derived from animal cancer tissue. This may interfere with mechanistic studies of cell behavior, making it difficult to distinguish biological effects caused by controlled experimental variables from those caused by Matrigel itself.

Spheroid and organoid models thus represent an appealing methodology to foster the ability of cells to self-assemble and grow organically. These models can include multiple cell types cultured together and promote a high degree of cell–cell interaction. The work of Onozato et al.^29^ is an example of how spheroid models can be used to model the edge and core effects of tumors. Organoids show good correlation with clinical outcomes.^30^ The reliability of the experiments depends also on the reproducibility with which the spheroids can be created (e.g., similarity in terms of dimensions). One typical disadvantage of 3D scaffold-free models in general is their inability to form vascular networks^43^ and the absence of perfusion. In such context, the continuous or pulsed flow of fluid over the cells induces physical stresses that could affect apoptosis^44^ and influence the readout of radiotherapy outcomes.

## 3 Scaffold-Based In Vitro Models for Radiobiology

The classification term “Scaffolds” refer to engineered structures and materials (typically polymers or hydrogels^45^) designed to reproduce some features of the natural ECM in order to promote physiological cell morphology, adhesion, and growth. Such microstructures are typically fabricated by employing manufacturing techniques,^46^ including, but not limited to, stereolithography, bioprinting, fused deposition modeling, inkjet printing, and two-photon polymerization (2PP) or other 3D fabrication approaches such as hydrogel self-assembly, electrospinning, gas foaming, or salt leaching.^47^ These microenvironments foster a 3D spatial distribution of cells similar to the natural tissue, overcoming the cell monolayer configuration of TCP models. The replication of ECM features such as rigidity, can lead to more in vivo-like expression of cancer proliferation and metabolism markers.^48^ Scaffolds also allow the replication of biological features such as vasculature and porosity at the microscale. Scaffold-based approaches enable as well better control of the cell density, by tuning their porosity, compared to spheroids or organoids, improving imaging (optical, electron, and immunofluorescent confocal microscopy) of cellular and subcellular components. Scaffolds’ features are also typically employed to guide cell network disposition and growth in 3D, which can be crucial for accurate alignment during radiation studies, especially in FLASH contexts where the beam spots can be very narrow in size.

Many different materials and fabrication methods have been employed to create scaffold-based models for radiobiology. Each of these methods has their respective advantages and disadvantages, and the outcome and biological end point studied serve as guidelines to select the most appropriate model. A comparative overview between scaffold-based, scaffold-free, and organ-on-a-chip models for radiobiology is presented in Table 2, while Table 3 provides an additional comparison in terms of the manufacturing methods and materials used in scaffold-based and organ-on-a-chip models.

**Table 2:**
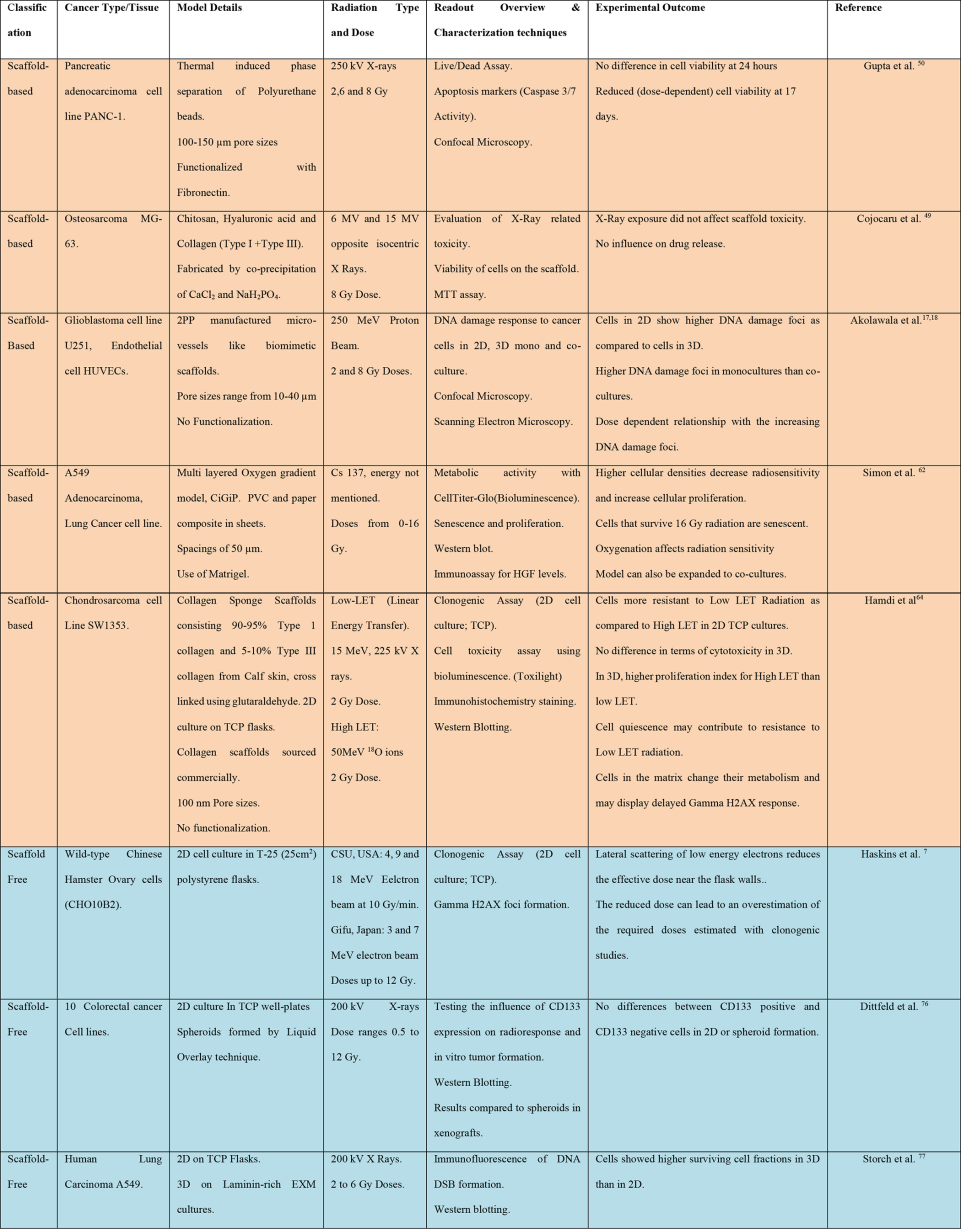

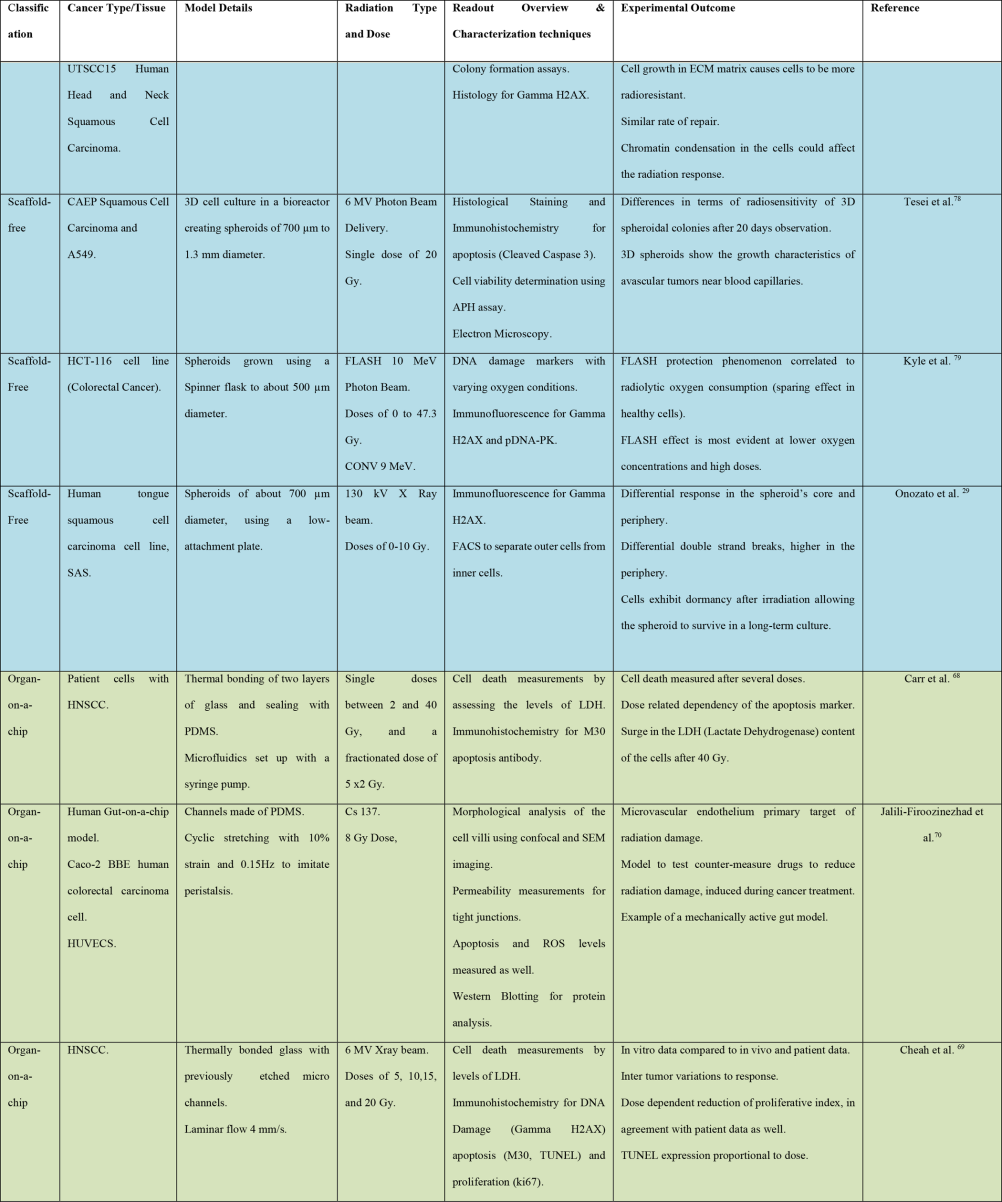
Summary of Various Scaffold-Based, Scaffold-Free, and Organ-on-a-Chip Models Used for Radiobiology

**Table 3:**
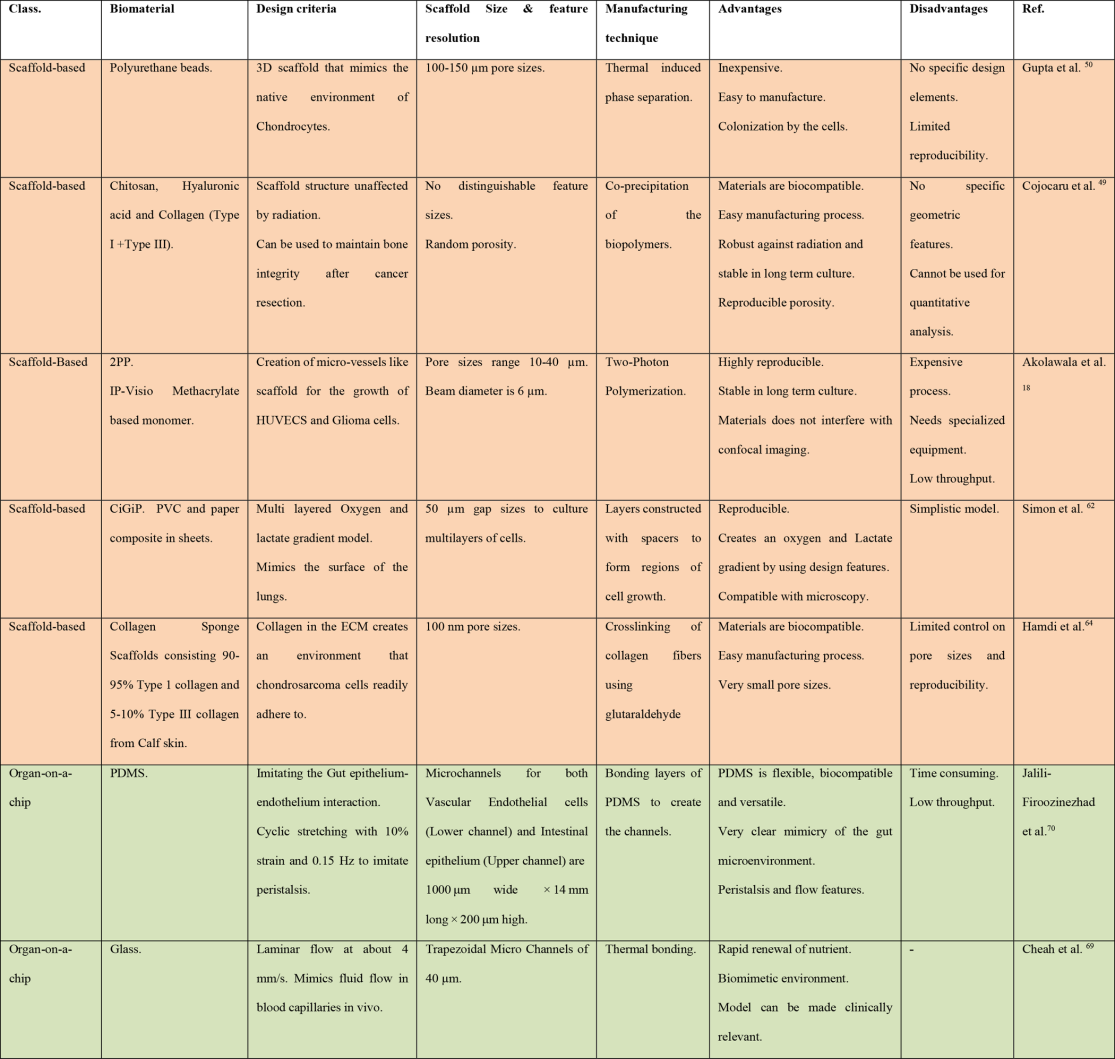
Comparison of Various Manufacturing Methods and Materials Used in Scaffold-Based and Organ-on-a-Chip Models for Radiobiology

Polymeric scaffolds are robust from a mechanical point of view (i.e., typically not prone to substantial swelling or shrinking in a cell medium) and can be created with a variety of biocompatible materials that allow their use as a tool for qualitative or quantitative analysis but also for applications as tissue grafts. Cojocaru et al.^49^ showed that the use of a chitosan-based polymer fabricated by the coprecipitation of CaCl_2_ and NaH_2_PO_4_ as a graft to replace cavities left behind after radiotherapy in bone tissue, could not only allow for the structural stabilization of the bone but also provide an environment that is not affected by radiation, and could be used for radioresistant cancer cell treatment using in situ drug release.

**Figure 3:**
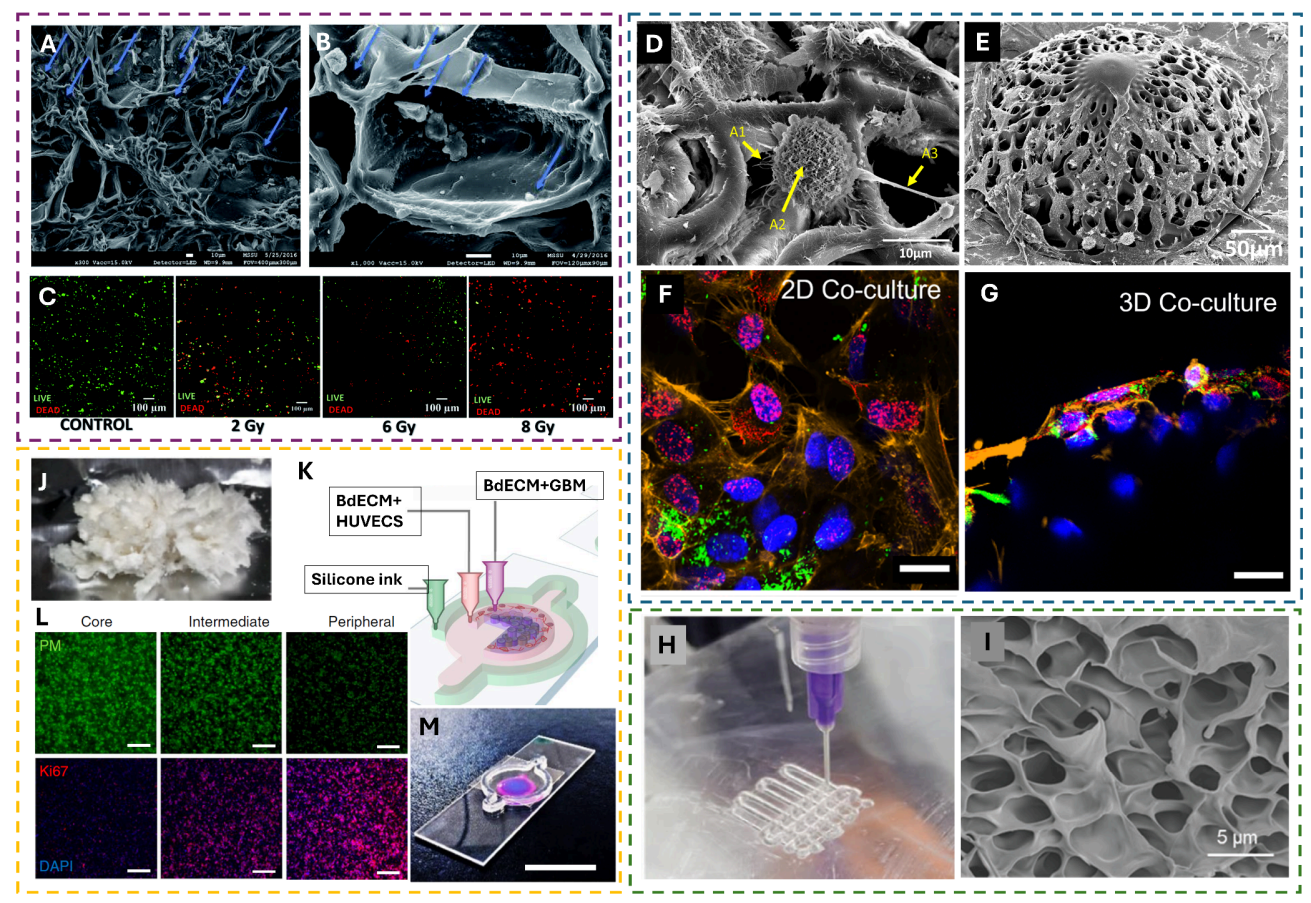
Examples of scaffold-based approaches to study the radiation response of cancer cells. (A and B) SEM images of PANC-1 cells in sections of uncoated PU scaffolds. The blue arrows point to cells growing on the scaffold (scale bar = 10 μm). Reproduced from ref (53). Available under CC-BY 3.0. Copyright 2018 Royal Society of Chemistry. (C) Effect of radiotherapy on PANC-1 cells in PU scaffolds 17 days post-treatment. Higher doses show a higher proportion of dead cells (scale bar = 100 μm). Reproduced from ref (51). Available under CC-BY 3.0. Copyright 2019 Royal Society of Chemistry. (D and E) 2PP-fabricated scaffolds cultured with HUVECs and U251 Glioma cell lines. The arrows indicate the microstructures on the surfaces of the cells that have been quantified. (F and G) 3D confocal images of the GBM cells/HUVECs in 2D and 3D coculture configurations after a 8 Gy proton irradiation dose. Red shows the Gamma H2AX foci, and green shows vWF used to distinguish HUVECs from GBM cells (scale bar = 20 μm). (D–G) Reproduced from ref (18). Available under a CC-BY License. Copyright 2023 Advanced Healthcare Materials published by Wiley-VCH GmbH. (H) Bioprinting of GAF scaffolds with embedded GBM cells. (I) SEM micrograph of the GAF scaffold without cells. (H and I) Reproduced with permission from ref (54). Copyright 2024 Wiley-VCH GmbH. (J) Decellularized porcine brain used to create the BdECM bioprinted construct. (K) Schematic of the glioblastoma-on-a-chip model made of BdECM bioinks with HUVECs and GBM cells used to create a compartmentalized structure. The silicone ink on the outer layer is gas-permeable to allow for the diffusion of gases to the cells. (L) Formation of a hypoxic core, as indicated by PM in green, and formation of a proliferative rim, as shown by K_i_-67 in red (scale bar = 200 μm). (M) Photograph of a mock glioblastoma-on-a-chip model using the laden HUVECs (magenta) and GBM cells (blue) bioink to show stratification of the layers (scale bar = 2 cm). (J–M) Reproduced from ref (55). Copyright 2019 Springer Nature Limited, under exclusive license.

Scaffold-based in vitro models have specific microarchitecture and geometries to mimic real tissue properties and feature higher reproducibility compared to spheroid and organoid models, although they are not as inexpensive and high throughput as TCP models due to the need of costly fabrication setups and relatively high fabrication time per sample. On the other hand, scaffold-based models enable long-term (weeks or months)^50^ postradiation studies for the evaluation of realistic treatment responses.^51,52^ The relative chemical stability of polymeric scaffold materials such as chitosan, polylactic acid (PLA), or polyurethane (PU; Figure 3A) makes this possible.

Parts A and B of Figure 3 show the growth of pancreatic ductal adenocarcinoma (PDAC) cell line PANC-1 on PU scaffolds.^53^ The scaffolds support the growth and proliferation of the PANC-1 cells for 29 days, without the formation of any necrotic region, and high cell viability with cellular self-assembly into dense clusters.^53^Figure 3C shows the PU scaffolds to evaluate the radiation response of the PANC-1 cells.^51^ The relationship between radiation and apoptosis is directly dependent on the dose, with the higher doses showing a greater number of dead cells. It was also reported that radiation-induced cell death for PANC-1 cells is only detected after 17 days in culture; thus, a platform that allows culture at such time lines is very informative.^51^ From a mechanobiology point of view, even if PLA and PU feature relatively high Young’s moduli (in the megapascal to gigapascal range), it is important to mention that the cell effective stiffness or the effective shear modulus that cells experience while interacting with 3D micro- or nanostructures depends on the architectural features of the biomaterial and is significantly softer than the stiffness of the material that the microstructures are composed of, as reported for mesenchymal stromal cells^56^ and neurons.^57^

These scaffold-based models can be suited to mimic other specific features of the tissue or cancer microenvironment such as the porosity of bone tissues,^49^ pancreatic ductal zone and compartmentalized architecture,^50^ or blood-vessel-like architecture to mimic part of the glioblastoma microenvironment.^17,18,58^ The use of high-resolution printing methods, such as 2PP,^59,60^ and the development of specific biomaterials (e.g., IP-Visio) featuring low intrinsic autofluorescence are, in particular, very promising for mechanobiology, in vitro disease modeling, and treatment. 3D microvessel-like scaffolds printed with IP-Visio and colonized by glioblastoma cells and human umbilical vein endothelial cells (HUVECs) are depicted in the micrographs of Figure 3D,E. The engineered glioblastoma (GBM) microenvironments reported by our group showed how 3D GBM models display an amount of DNA damage foci, upon exposure to conventional proton radiation, lower than 2D GBM models (in line with the comparison between natural GBM tissue versus 2D models) and that endothelial cells have a direct effect on GBM radioresistance.^18^ The difference in terms of amount of DNA damage foci between 2D and 3D coculture models can be qualitatively seen in Figure 3F,G and has been quantitively assessed as well. This fabrication method has broad applications due to its versatility in terms of feature resolution and a high degree of design control. The employed photo-cross-linkable materials are stable in cell medium, are compatible with multiple cell types, and remain stable upon exposure to radiation. The same microfabrication technique (2PP) was employed to show how microscaffolds, featuring different Young’s moduli and stiffness gradients, enable cancer cell invasion in the presence of softer architectures, while the introduction of 3D stiffness “weak spots” boosts the rate at which cancer cells invade the scaffolds.^61^ Scaffold-based approaches were also used in specific systems to create an oxygen and lactate gradient in the cell medium through a perforated acrylic plate, as demonstrated by Simon et al. in their work.^62^ Their model was compatible with fluorescent microscopy, and they were able to infer that the O_2_ gradient, lactate gradient, and cell density can affect how the cells respond to radiation, showing that decreasing levels of oxygen can reduce cellular proliferation of nonsmall cell lung cancers and increase their radioresistance.

In the presence of 2D TCP models, the effectiveness of radiation response is measured often by the “gold standard” clonogenic assays in which the radiation lethality is defined as the reduced reproductive capacity of the cells. For 3D-engineered microenvironments, however, this standard does not exist yet.^63^ In particular, there are challenges associated with the lysis of cells adhering only on 3D scaffolds, the extraction of the cell lysate, and the establishment of standard protocols to compare the outcomes of different radiation modalities, energies, materials, design, and fabrication parameters.^63^ An alternative is reported in other recent works, which employed confocal immunofluorescence imaging instead and extensive morphological analysis and characterization.^18,64^

To further study the mechanisms of radiation responses of cancer, 3D bioprinting provides another appealing alternative. Bioprinting is a process by which live cells are encapsulated within a biomaterial and then printed into a desired geometry. Liu et al.^54^ in their work report the use of a gelatin alginate–fibrinogen (GAF) hydrogel system as a 3D material in which GBM cells are embedded and printed into woodpile scaffolds, as depicted in Figure 3H. Figure 3I shows a magnified micrograph of the microstructure of the material. These matrices foster cellular–biomaterial interaction with a highly controlled spatial distribution of the cells in the material. The authors showed that encapsulation of the cells in the bioink does not significantly affect the cell viability.^54^ The use of this method allows one to create a scaffold featuring a 3.2 kPa Young’s modulus, which is comparable to the brain ECM, ranging between 0.1 and 1 kPa.^65^ After the bioprinted constructs were exposed to X-ray radiation doses of 0, 2, 4, 6, and 8 Gy, it was reported that the 3D models featured increased radioresistance and higher cell survival than the corresponding 2D models. In another example, Yi et al.^55^ employed a decellularized porcine brain matrix (BdECM; Figure 3J) to create a bioprinted architecture. The design, as shown in Figure 3K, employs the BdECM gel to culture glioblastoma cells and HUVECs in concentric rings to mimic the cross section of the tumor with a gas-permeable silicone outer ring and allow gas exchange. They observed, as reported in Figure 3L, the formation of a hypoxic core [indicated by pimonidazole (PM)] and a highly proliferative index on the rim of the construct (indicated by K_i_-67). Figure 3M is a photograph of a mock glioblastoma-on-a-chip model showing the concentric rings printed with the BdCEM bioink. The design of the model featuring a central bioprinted GBM core creates an oxygen gradient from the rim to the center, facilitated by the presence of the gas-permeable silicone layer. The formation of such an oxygen gradient and the cellular heterogeneity in the model is highly representative of the in vivo tumor model, and the use of materials extracted from biological sources promotes cell–cell and cell–matrix interactions. The bioprinted glioblastoma-on-a-chip models were then subjected to chemoradiotherapy by following the treatment protocols of the patients from whom the cells were derived. The cells were exposed to γ radiation, and a positive correlation between the outcomes of the patients and the cell survival outcomes of the on-chip models was observed.

3D scaffold-based models overcome a significant problem of organoid and spheroid models (3D scaffold-free models), which is reproducibility. These models can use a larger variety of cell types because they can be designed and fabricated to the dimensional requirements of the cells, are mechanically robust, and can be employed for the use of coculture.^18^ While they do not have fluid-flow features, such models can be integrated within organs-on-chips or a pump system. Optimizing the parameters of fabrication to successfully integrate these models within flow would require expertise and insights from engineering and materials sciences but can lead to reproducible cellular models. Furthermore, these models can be optimized for the assays in which they will be employed, by incorporating specific features such as transparency and nonautofluorescence for immunofluorescence-based assays, as well as surface treatments for protein-, DNA-, and RNA-based analysis. An important aspect to consider is that these models typically require specialized microfabrication equipment (depending on the technique) and expertise from cross-domain collaborations. Finally, even though the use of synthetic polymers in the models can have advantages in terms of mechanical robustness and reproducibility, additional efforts are needed to further develop semisynthetic or natural hydrogel materials better mimicking the ECM with which cells interact in vivo.

## 4 Organ-on-a-Chip In Vitro Models for Radiobiology

Among all of the models that we have discussed so far, either 2D, 3D, scaffold-based, or scaffold-free, a major limitation is the inability to model blood or fluid flow around the cells. These “dynamic” features contribute, among other things, to mechanical stress both on the extra cellular environment and on the cells themselves. Microfluidic flows also allow the perfusion of biochemical cues, oxygen, and nutrients within the cells. Organ-on-a-chip (OOC) models^66^ can overcome this limitation. They typically involve a 2D or 3D cell culture configuration and a fluid flow featuring biologically relevant flow rates and pressures. Poly(dimethylsiloxane) (PDMS)^67^ is a widely used material for the fabrication of these chips due to its flexibility, transparency, biocompatibility, and relatively low Young’s modulus.

**Figure 4:**
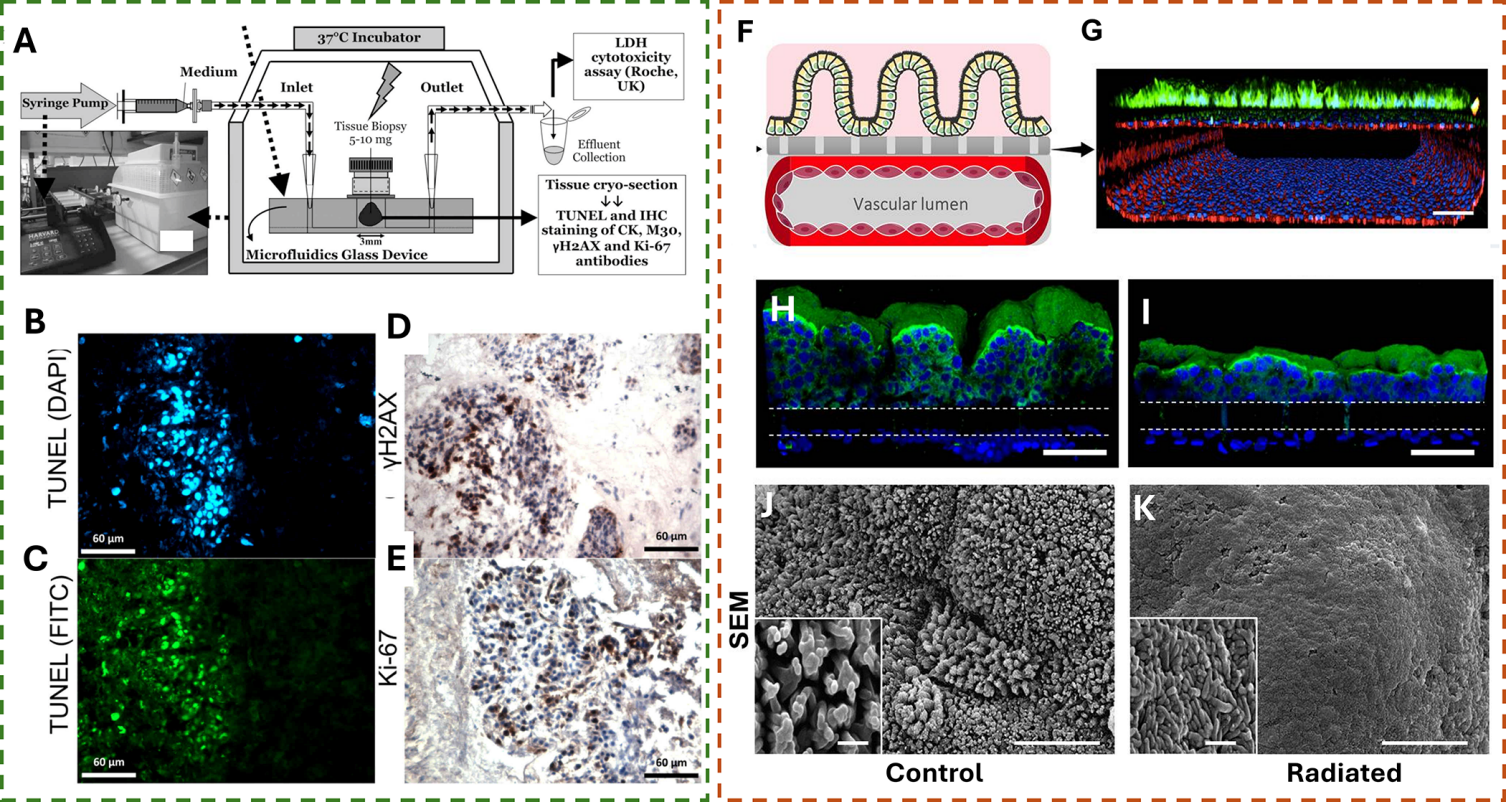
Examples of OOC models used for the cancer radiation response. (A) Schematic diagram of a microfluidic culture set up for the study of the HNSCC response to radiation. A syringe pump is connected to the microfluidic device, which provides continuous flow. (B–E) Representative images of serially sectioned lymph node tissue containing tumor metastases incubated in the microfluidic device, visualized at 400× magnification 24 h after being exposed to 5 Gy radiation treatment. (B and C) TUNEL assay and (D and E) immunohistochemistry staining of γH2AX and K_i_-67 of HNSCC cells exposed to 6 MV X-rays (scale bar = 60 μm). (A–E) Reproduced with permission from ref (69). Copyright 2017 Spandidos publications. (F) Schematic showing an OOC with the top section housing intestinal epithelial cells showing villi-like formations and the lower chamber with a hollow lumen, made of endothelial cells, that allows fluid flow. (G) Representative immunofluorescence confocal 3D reconstruction visualizing a cross section of the gut-on-a-chip device (scale bar = 100 μm). (H) Cross-sectional 3D view of the endothelium–epithelium layers (scale bar = 100 μm). (I) Shortening of the villi by radiation-induced damage (scale bar = 100 μm). (J) SEM micrograph images of the formed villi structures [scale bar = 1 μm (inset) and 10 μm (low magnification)]. (K) Villi structures smoothed out after irradiation (scale bar = 1 μm (inset) and 10 μm (low magnification)]. (F–K) Reproduced with permission from ref (70). Available under a CC-BY license. Copyright 2018 Springer Nature.

While not extensively employed for the response of cancer cells to radiation, the OOC models hold great promise for radiobiology studies. Carr et al. in their work^68^ used head and neck squamous cell carcinoma (HNSCC) tissue biopsies from patients in a OOC model. The microfluidic device was manufactured between two layers of glass thermally bonded together. The tissue is added to the central well and sealed. The use of a syringe pump created a flow of 2 μL min^–1^ to maintain tissue viability. They then exposed the model to 6 MV photon radiation at clinically relevant doses of 2 and 40 Gy and a fractionated course of 5 × 2 Gy. They demonstrated that such a model could be used to study radiation responses in HNSCC cells over a period of days after irradiation.^68^ They also found that the cells used in the OOC model showed increased levels of lactate dehydrogenase (LDH), which leads to cell death. The LDH levels were measured from the effluent medium from the chip. Cheah et al. used a similar model with 6 MV X-rays to study different end points for HNSCC such as DNA damage and apoptosis assays^69^ (Figure 4A). Their outcomes showed a dose-dependent increase in the Gamma H2AX expression in the cells undergoing radiation and a decrease in the expression of proliferation indicated by K_i_-67. They also observed an increase in the TUNEL (apoptosis) expression (Figure 4B,C).

Representative images of Gamma H2AX and K_i_-67 histological staining are shown in Figure 4D,E and correlate to in vivo and patient data, thus providing a viable alternative to using xenograft models for such studies, which can take up to 6 months to generate.^69^ Jalili-Firoozinezhad et al.^70^ in their work modeled a gut-on-a-chip using PDMS (Figure 4F–K) and reproduced an endothelium–epithelium interface of the intestinal tissue. The use of an OOC allowed for the creation of a functional “blood vessel” enabling the flow of nutrients through its lumen, required by the cells, along with peristaltic cycling that is essential in a gut model. Upon exposure of the model to 4–8 Gy of γ radiation, they were able to observe disruption caused by the radiation exposure on gut cells and, in particular, on the endothelium, as depicted in Figure 4H,I, where the characteristic villi of the intestinal cells are flattened due to radiation damage. The scanning electron microscopy (SEM) micrographs in Figure 4J,K clearly show this flattening, which in the human gut would reduce the absorption of nutrients from food. OOCs have also been used to create functional models of very complex regions of the brain such as the blood–brain barrier (BBB) and to study the response of the BBB to glioma cells,^71^ thus representing an interesting tool to study the in vitro radiation response of the BBB.

OOC models can recreate physiologically relevant flow, cell interactions, and regions within the chip in which cells can perform specific functions. Gas permeability, the creation of oxygen gradients within the chips, nutrient flow, and mechanical stimuli are typical features in such models that can involve various cell types, as well as their interactions with each other and the ECM, simultaneously. On the other hand, OOC models can require a long period of design and development to successfully include all of the above-mentioned features. OOC models can also be difficult to handle and to be kept sterile due to their many parts, pumps, and tubing, thus increasing the required considerations for radiation experiments.^43,72^ Finally, even though these types of models feature high fidelity and biological relevance, they are also affected by a significantly lower throughput^73^ compared to TCP approaches, for instance.

## 5 Conclusions and Future Focus

Cancer is one of the first causes of death worldwide.^74^ Among cancer types, some of them, such as glioblastoma (the most aggressive brain cancer) or pancreatic cancer, do not yet have a cure and/or have a low survival rate. This means that current treatments for these cancers, typically involving surgery, chemotherapy, and/or radiotherapy, are still ineffective. One of the main reasons behind the ineffectiveness of such treatments is the huge gap between in vitro cancer models and the in vivo cancer tissue configuration, which unavoidably leads to possible mismatches between what is observed in preclinical studies and clinical ones. TCP models and their associated assays have formed the foundational understanding of cellular radiation response, but a key question is whether such cell survival in vitro models can represent clinical tissue outcomes.^43^ The persistent failure to translate promising drug/treatment candidates from laboratory to clinical use highlights the limited relevance of the current state-of-the-art.^73^ There is also a large body of evidence, as we describe here and elsewhere, about the discrepancy between the expected and actual radiotherapy outcomes, which can be partially attributed to the transition from a “2D setting” to a “3D tissue environment”. 3D environments have been shown to impact cell growth, proliferation, cell fate, and increased radioresistance.^43,75^ Radiotherapy studies within 3D models is currently under-researched, and because of the aforementioned impact, the expansion toward more 3D ECM-like microenvironments is an urgent need for the development of physiologically relevant, reproducible, patient-derived models. Our current knowledge indicates that 3D models feature increased radioresistance through (i) increased stemness expressions, preserving the abilities of cancer cells to regenerate, (ii) the TME and mechanobiological cues contributing to the radioprotection of the cells, (iii) the presence of noncancer cells (such as stromal cells) around the tumor cells that lead to the activation of cellular pathways, making cancer cells more robust to radiation. These are points of attention that currently have a preliminary body of evidence and that need to become avenues for further research in the field. In this Perspective, we highlight recent efforts in the development of engineered models employed in the field of radiobiology and compare their pros and cons. In order to improve the biofidelity of such models, it is imperative in our opinion to further propel the development of hybrid approaches exploiting the best features of scaffold-free, scaffold-based, and organ-on-a-chip models. Indeed, while scaffold-based models provide precise topographic and biomechanical cues, they often lead to the formation of relatively small (monoculture) cell networks, which do not recapitulate the complexity of the TME. To overcome this limitation, we envision that, with the continuous improvement of 3D (bio)printing techniques, future scaffold-based models shall be merged with scaffold-free ones, by combining scaffold technology with organoid technology. In this way, it will be possible to integrate co-, tri-, or multiculture models (involving cancer, healthy, and immune cells), favor cell–cell interaction, and enable the development of controlled, reproducible tissue-like culture. Further, to fully mimic the natural tissue, it will be of paramount importance also to add “dynamic” features by building these hybrid constructs within microfluidic organ-on-a-chip devices in order to control the perfusion of nutrients and oxygen. The inclusion of perfusion and multicellular tissue models is challenging because of the varied approaches and lack of standardization. The research must thus not only focus on the development of these models but also consider comparability and validation of the models. In such a context, researchers should develop systematic ways of defining standard criteria to facilitate the definition and development of disease-relevant assays to screen out irrelevant cell-based models, following the example of Horvath et al.^73^ Finally, it will also be imperative to adapt the current DNA damage, apoptosis, proliferation, and clonogenic assays (compatible nowadays mostly with TCP models) to this new class of engineered microenvironments in order to deliver tangible radiobiology benchmark tools that can pave the way toward personalized cancer medicine. One way to employ the described models for personalized therapy could involve the use of minimally invasive biopsies from a patient’s cancerous tissue. Upon mechanical and enzymatic dissociation, the cells could be then cultured within the specific engineered microenvironments to foster the formation of reproducible cell-scale or tissue-scale networks, expose them to a set of different radiation doses, and evaluate the amount of DNA damage response as well as clonogenicity, which could guide the choice of the most appropriate personalized (radio)treatment. In summary, 3D-designed and -engineered models are one arm of a larger cohesive effort to create precise, accurate, clinically relevant and reliable translational methods.^73^

## Abbreviations

2PP: two-photon polymerization
2D: two-dimensional
3D: three-dimensional
BdECM: brain-decellularized extracellular matrix
CSCs: cancer stem cells
DSB: double-strand breakage
ECM: extracellular matrix
EdU: 5-ethynyl-2′-deoxyuridine
GAF: gelatin alginate–fibrinogen
GBM: glioblastoma
HNSCC: head and neck squamous cell carcinoma
IR: infrared
LDH: lactate dehydrogenase
OOC: organ-on-a-chip
PDAC: pancreatic ductal adenocarcinoma
PLA: polylactic acid
PU: polyurethane
SAS: squamous carcinoma cells
SEM: scanning electron microscopy
SOB: spread-out Bragg peak
TCP: tissue culture plastic
TME: tumor microenvironment
